# A cross-sectional analysis of popular hidradenitis suppurativa content on TikTok

**DOI:** 10.1016/j.jdin.2021.09.004

**Published:** 2021-10-11

**Authors:** Rafey Rehman, Marwa Saad, Farhan Huq, Sandra Oska, Darius Mehregan, Steven Daveluy

**Affiliations:** a Oakland University William Beaumont School of Medicine, Rochester, Michigan; b Department of Dermatology, Wayne State University, Dearborn, Michigan; c Department of Dermatology, Henry Ford Health System, Detroit, Michigan

*To the Editor:* As of January 2021, TikTok is an application with about 689 million monthly users, where users can upload videos ranging from 2 seconds to 1 minute.^1^ However, TikTok recently announced that users could upload videos for up to 3 minutes. This enables TikTok to be used as a video-based educational modality, similar to YouTube, to improve literacy for diseases with a delayed diagnosis, such as hidradenitis suppurativa (HS).^2^ Although TikTok was primarily created for entertainment, it is important to analyze HS information quality if viewers use it for education. The purpose of this study was to analyze TikTok's popular HS content quality and identify areas to improve patient outcomes.

We searched TikTok for videos tagged with #hidradenitissuppurativa on April 1, 2021, and analyzed the top videos returned by the TikTok search algorithm to include 100 videos that met the inclusion criteria. Videos that were non-English, unrelated to HS, or duplicates were excluded. Video characteristics were collected, and the content quality was determined by two independent reviewers using DISCERN, a validated 16-item questionnaire that assesses consumer health information quality based on criteria such as references, treatment risks and benefits, and information relevance using a scale of 1 (poor)–5 (excellent).^3^

A total of 119 videos were screened to identify our target of the top 100 videos, which had a combined 1,098,036 likes and 23,533 comments. The videos had a mean DISCERN score of 1.77, with high interrater reliability (Cohen's Kappa > 0.8). Stratified by the content creator, there were 84 videos (84%) by non-physicians with a mean DISCERN score of 1.63 and 13 videos (13%) by physicians with a mean DISCERN score of 2.65 (Table I). Content creators received particularly low DISCERN scores on items involving publication sources and dates, as well as treatment risks and benefits. Fig 1 provides an overview of the average DISCERN item breakdowns.

**Table I:** Overview of hidradenitis suppurativa content on TikTok

	No. of videos (n = 100) (%)	Mean no. of likes	Mean no. of comments	Mean DISCERN scores
Content creator				
Non-physician	83 (83)	4126	117	1.63
Physician	13 (13)	57,559	1043	2.65
Private company	4 (4)	806	41	1.38
Gender				
Female	86 (86)	4682	125	1.67
Male	13 (13)	53,104	969	2.24
Other designations	1 (1)	380	24	1.67
Physician specialty				
Dermatology	12 (12)	56,990	926	2.70
Family medicine	1 (1)	64,400	2449	2.00
Video types				
Personal experience	59 (59)	2686	118	1.55
Educational HS content	15 (15)	37,952	670	2.43
Home remedies	12 (12)	5299	105	1.96
Treatment advertising	11 (11)	24,751	421	1.82
Other	3 (3)	23,070	163	1.07

**Fig 1:**
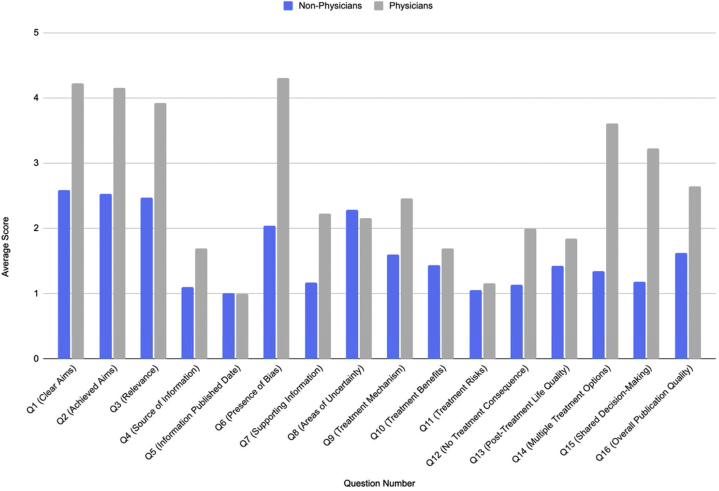
Average DISCERN item scores among physicians vs non-physicians.

Our analysis revealed that physician videos had a higher mean DISCERN score (2.65) than non-physician videos (1.63) (*P* < .01). Although DISCERN scores among physicians were higher than non-physicians, content creators can generally improve video quality by discussing treatment risks and benefits, providing content references, and consulting local physicians. Given TikTok's recent upgrade to video length, it is now more feasible to use this platform to disseminate information. However, high quality videos may not correlate linearly with viewership, and it is important to incorporate trending themes (such as songs and dances) to increase video popularity.^4^

Study limitations include limited generalizability to different periods given the cross-sectional design and TikTok turnover rate. Although DISCERN was validated to assess written educational material quality and does not assess content accuracy, it has recently been used to appraise social media video quality.^5^ A validated tool to assess social media content should be developed, as the exact link between high DISCERN scores and viewership is unclear. Although TikTok was not designed to be an educational platform, it may be worth the effort to improve video quality if viewers use it for such purposes.

## Conflicts of interest

None disclosed.
